# Difference in the net value of ecological services between natural and artificial forests in China

**DOI:** 10.1111/cobi.13293

**Published:** 2019-04-08

**Authors:** Shixiong Cao, Junze Zhang, Wei Su

**Affiliations:** ^1^ School of Economics Minzu University of China No. 27, Zhongguancun South Street Haidian District Beijing 100081 P.R. China; ^2^ Faculty of Geographical Science Beijing Normal University No. 19, Xinjiekouwai Street Haidian District Beijing 100875 P.R. China; ^3^ College of Economics and Management Beijing Forestry University No. 35, Qinhuadong Road Haidian District Beijing 100083 P.R. China

**Keywords:** afforestation, cost analysis, ecological restoration, sustainable development, análisis de costos, desarrollo sustentable, repoblación forestal, restauración ecológica

## Abstract

Land degradation is a global problem that seriously threatens human society. However, in China and elsewhere, ecological restoration still largely relies on a traditional approach that focuses only on ecological factors and ignores socioeconomic factors. To improve the effectiveness of ecological restoration and maximize its economic and ecological benefits, a more efficient approach is needed that provides support for policy development and land management and thereby promotes environmental conservation. We devised a framework for assessing the value of ecosystem services that remain after subtracting costs, such as the opportunity costs, costs of forest protection, and costs for the people who are affected by the program; that is, the net value of ecosystem services (NVES). To understand the difference between the value of a resource and the net value of the ecosystem service it provides, we used data on VES, timber sales, and afforestation costs from China's massive national afforestation programs to calculate the net value of forest ecosystem services in China. Accounting for the abovementioned costs revealed an NVES of ¥6.1 × 10^12^ for forests in 2014, which was 35.9% less than the value calculated without accounting for costs. As a result, the NVES associated with afforestation was 55.9% less than the NVES of natural forests. In some regions, NVES was negative because of the huge costs of human‐made plantations, high evapotranspiration rates (thus, high water opportunity costs), and low forest survival rates. To maximize the ecological benefits of conservation, it is necessary to account for as many costs as possible so that management decisions can be based on NVES, thereby helping managers choose projects that maximize both economic and ecological benefits.

## Abstract 2

*Article impact statement*: Accounting for the effects of costs on the value of ecosystem services provides a net value that more strongly supports policy development.

## Abstract (es)

Diferencias en el Valor Neto de los Servicios Ecológicos entre los Bosques Naturales y Artificiales de China

**Resumen**

La degradación del suelo es un problema global que amenaza seriamente a la sociedad humana. Sin embargo, en China y en otras partes, la restauración ecológica todavía depende en gran parte de una estrategia tradicional que se enfoca solamente en los factores ecológicos e ignora los socioeconómicos. Para aumentar la efectividad de la restauración ecológica y maximizar sus beneficios ecológicos y económicos se necesita de una estrategia más eficiente, la cual proporcione un respaldo para el desarrollo de políticas y el manejo del suelo y así promueva la conservación ambiental. Diseñamos un marco de trabajo para la evaluación del valor de los servicios ambientales que permanecen cuando se restan los costos, como los costos de oportunidad, los costos por la protección del bosque, y los costos para quien es afectado por el programa; es decir, el valor neto de los servicios ambientales (NVES, en inglés). Para entender la diferencia entre el valor de un recurso y el valor neto del servicio ambiental que proporciona usamos datos del valor del servicio ambiental, ventas de madera y costos por repoblación forestal tomados de los enormes programas nacionales de repoblación forestal de China para calcular el valor neto de los servicios ambientales de los bosques en ese mismo país. Cuando consideramos los costos antes mencionados, se reveló un NVES de ¥6.1 × 10^12^ para los bosques en 2014, el cual fue un 35.9% menos que el valor calculado sin considerar los costos. Como resultado, el NVES asociado con la repoblación forestal fue 55.9% menor que el NVES de los bosques naturales. En algunas regiones, el NVES fue negativo debido a los altos costos de las plantaciones artificiales, las tasas altas de evapo‐transpiración (por lo tanto, altos costos de oportunidad hídrica) y las tasas bajas de supervivencia de los bosques. Para maximizar los beneficios ecológicos de la conservación es necesario tomar en cuenta el mayor número posible de costos para que las decisiones de manejo puedan basarse en los NVES, por lo que ayudarían a los administradores a elegir proyectos que incrementen los beneficios sociales y económicos.

## Introduction

Since the end of the 20th century, the number of studies of ecosystem services has grown exponentially (e.g., Costanza et al. 1997; Gómez‐Baggethun et al. 2010; de Groot et al. 2012). Many scholars have studied and discussed the definition, classification, and methods of evaluating ecosystem services extensively (e.g., Boyd & Banzhaf 2007; Wallace 2007; Fisher & Turner 2008; Pandeya et al. 2016) and have debated the economics of ecosystem services (Farber et al. 2002; Farley 2012). Although the definitions and classification of ecosystem services in the Millennium Ecosystem Assessment (MEA 2003) have been widely quoted, the goal of this group was to clarify the relationship between ecosystems and human well‐being by forecasting future ecosystem changes; the group does not provide an appropriate framework for valuing current ecosystem services. This has led to differences among researchers in their assessment of the value of current ecosystem services, even for the same ecosystem in the same region (Barbier 2010; Costanza et al. 2014).

Although the benefits of nature to households, communities, and economies are generally known, there are also large costs associated with ecosystem services, and performing cost–benefit analyses of ecosystem services provides support for decision making and policy development (NRC 2005; Naidoo et al. 2006). Cost–benefit analyses of ecosystem services have achieved some important results (Wegner & Pascual 2011) in, for example, the fields of water conservation (Immerzeel et al. 2008), marine aquaculture (Zheng et al. 2009), wetland construction and conservation (Chen et al. 2009), forest restoration (Birch et al. 2010), and biodiversity conservation (Peh et al. 2013). However, most researchers accounted for only part of the cost (Pandeya et al. 2016). By failing to account for as many costs as possible, such studies exaggerate the perceived benefits provided by an ecosystem, thereby misleading managers about the requirements for maintenance and restoration of the ecosystem to provide these benefits and reducing the likelihood that management will be successful (Birch et al. 2010; Kareiva et al. 2011).

It is important to account for costs in analyses of the value of ecosystem services (VES) so as to identify the true net benefits of projects (Goldstein et al. 2008). By improving their understanding of costs, managers will obtain a more holistic picture of the land they manage and will thereby have an opportunity to increase the effectiveness of their environmental protection projects (Chen et al. 2009). When those who make management decisions, such as the choice between natural recovery and afforestation or between forest protection and timber production, are the primary beneficiaries of an ecosystem service, focusing on their benefits may ignore important costs for the stakeholders affected by their decisions. These costs can only be accounted for when stakeholders are consulted and informed of the direct costs (e.g., management expenses) and indirect costs (e.g., opportunity, risk) of proposed management decisions. Thus, an analysis of a decision's optimal benefit level, based on a holistic assessment of the costs and benefits for all stakeholders, is critical. To improve the effectiveness of ecological restoration and maximize economic and ecological benefits, it is necessary to move toward a more efficient approach that provides support for policy development and land management that will promote environmental conservation.

Toward this goal, we developed an approach that accounts for additional costs, such as opportunity costs, that are omitted from traditional VES research. We combined previously published VES data, including earnings from timber sales, with data on afforestation and forest protection costs to provide a more realistic assessment of the true benefits obtained from China's afforestation program. The results should help Chinese managers design more effective ecological restoration policies.

## Methods

To compare the potential net value of ecosystem services (NVES) under China's national afforestation policy with the NVES of natural forests, we used data from Niu et al. (2012) and Wang et al. (2017) on the value of forest ecosystem services. Wang et al. (2017) used data from the seventh national investigation of forest resources and consecutive observations from 2005 to 2009 by personnel of the long‐term Ecological Research Station (affiliated with the Chinese Forest Ecosystem Research Network) and the methods of the State Forestry Administration to determine the value of forest ecosystem services in China.

The main ecosystem services in their study were water conservation, biodiversity protection, carbon fixation, oxygen generation, soil conservation, maintenance of soil fertility, air purification, and nutrient accumulation (Niu et al. 2012). Niu et al. (2012) did not consider the value of forest resources, forest byproducts, and the value of the forest land. To account for these values and obtain a more holistic estimate of the value of the goods (forest resources and byproducts) obtained from forest ecosystems, we used data on the allowable harvest in these areas taken from management plans and estimated the potential value from sales of timber based on mean national wood costs in 2014 (SFA 1987–2014). Although timber harvesting is not permitted in most of China, some harvesting is allowed under regional forest management plans. We also obtained data on the value of the land (see below).

When we estimated the benefits (i.e., goods and services) provided by a forest ecosystem in a given situation, we also considered the costs (*C*) associated with these benefits, which include direct costs (*C*_d_), opportunity costs (*C*_o_), and risk costs (*C*_r_) associated with these benefits. We defined an NVES that represents the real benefits (including income) after accounting for these costs:

(1) net  benefits = benefits −C,

(2) NVES = VES −C, and

(3) $$C=C_{d}+C_{o}+C_{r},$$

where benefits are the profits from the sale of timber (the only economic value for forest products for which data were available), VES is the value of ecosystem services from Niu et al. (2012) and Wang et al. (2017), *C*_d_ is the direct cost of ecological conservation and restoration, *C*_o_ is the opportunity cost of using resources for afforestation rather than for alternative uses (e.g., using land for residential construction, using water for irrigation of crops), and *C*_r_ is the cost entailed by risks. We defined these risks as the actual management costs to protect against insects, diseases, and wildfire.

We converted all values to 2014 values based on the government's official mean annual inflation rate of 4.9% for the study period. To simplify the calculations, we defined *C*_o_ as the opportunity cost that results from not using the land for other purposes, which represents land rent (*C*_l_), and from not using the water (*C*_w_) for other, nonforest, economic objectives:

(4) $$C_{o}=C_{l}+C_{w}.$$

We obtained the average land rent in every province from a Chinese real‐estate market database (http://www.tdzyw.com). Our approach is coarse grained, and we hope future researchers will use finer‐grained data and the values for additional combinations of land uses.

The risk cost (*C*_r_) is what must be paid for forestry management to prevent natural disasters, including wildfires and outbreaks of plant diseases and insect pests. We based *C*_r_ on government statistical data on expenditures for the control of insects, diseases, and wildfire (SFA 1987–2014).

We obtained data from 1952 to 2014 on the area of natural forests and afforestation in each Chinese province, including provincial‐level cities (e.g., Beijing), from China's annual forestry statistical yearbooks, and China's first to eighth national forest resource inventory bulletins (SFA 1987–2014). The amount of surviving forest in a given year was obtained by dividing that year's existing area of forest in afforestation areas by the cumulative area of afforestation before that year. We multiplied this area of surviving forest by the water consumption per unit area to calculate the total water consumption. Forests that had serious mortality after afforestation underwent a variety of successional processes. Some underwent desertification, whereas others degraded into other vegetation types (e.g., grassland with sparse trees). It is also possible that some areas were converted to farmland or grazing areas by local residents. Because China's natural forests are generally mature, stable forests, we assumed survival of natural forests was 100%. If better data on survival trends for these forests becomes available at provincial or subprovincial scales, our analysis could be updated to account for differences in survival among different natural forest types and ages across China.

Because many of the areas where afforestation has been conducted are arid to semiarid, water is a precious resource in these areas. We therefore used potential evapotranspiration to represent water consumption by human‐made and natural forests based on 7 previously developed evapotranspiration models. All 7 models were tested by Chen et al. (2014) to confirm their ability to reliably estimate potential evapotranspiration under Chinese conditions.

Where reliable data were available on water costs within a province, we used them in our analysis. Where such data were not available, we used existing data to estimate the cost, such as data from the South‐to‐North Water Diversion Project (Zhang et al. 2016, 2017). Because prices increase as the scarcity of a resource increases, we assumed the cost of water increases as supply decreases in a given region. To estimate the different costs, we used the following equation:

(5) V=b+a×P,

where *V* is the value of the resource (e.g., water), *P* is the per capita water availability in the province, and *b* and *a* are curve‐fitting parameters. We multiplied the available water (i.e., annual precipitation [SCA 1986–2014]) by the area of the province to obtain the total available water. We then divided that amount by the provincial population to obtain the per capita water resource value.

To estimate these coefficients, we assumed the water price in Beijing (per capita water availability of 408 m^3^), which has the most expensive water in China, was Ɏ1.2 m^−3^ in 2014 based on data from the South‐to‐North Water Diversion Project (Zhang et al. 2016, 2017). We then assumed the water price on the Tibet Plateau, which has the highest per capita water availability in China (162,667 m^3^), represented the lowest price in China, and this value was Ɏ0.17 m^−3^ (Wang et al. 2009). We used Eq. (5) to linearly interpolate the value between these 2 points as a function of the per capita water availability. The results were *a* ≈ 6.351 × 10^−7^ and *b* ≈ −1.197.

Although it is possible that a different equation form would be more realistic than the simple linear equation in Eq. (5), defining the optimal equation form was beyond the scope of this paper. Many factors affect water prices, and accounting for all of them is a complicated challenge that should be solved in future research. A better solution would be to use actual data on water prices as these data become available or to obtain enough data on prices and water availability to support regression analysis to determine the optimal equation form.

## Results

China's natural and artificial forests covered 69.2 × 10^6^ and 147.9 × 10^6^ ha, respectively, in 2014, and had a total annual VES of Ɏ9.6 × 10^12^ (Table 1). This included annual values of Ɏ6.4 × 10^12^ and Ɏ3.2 × 10^12^ for natural and artificial forests, respectively. The annual VES per unit area was similar for natural and artificial forests, Ɏ43.2 × 10^3^ and Ɏ46.1 × 10^3^ ha^−1^, respectively. However, after accounting for the costs, including *C*_d_ (conservation investments), *C*_o_ (water consumption due to potential evapotranspiration, land rent), and *C*_r_ (management to prevent natural disasters), annual NVES totaled Ɏ6.1 × 10^12^ RMB for forest ecosystem services in 2014, which is 35.9% less than the value calculated without accounting for costs. As a result, the annual net benefit of the afforestation areas was Ɏ15.9 × 10^3^ ha^−1^, which is 55.9% less than that of natural forest. Even when we included income from timber production (China's policy prohibits timber harvesting in most of the country), the net benefits were only Ɏ6.4 × 10^12^ for China's forest ecosystem (both forest types combined) in 2014, which is 33.0% less than the VES.

**Table 1:** The value of ecosystem services calculated without accounting for costs (VES), the costs of providing these services (*C*_d_, direct costs; *C*_o_, opportunity costs; *C*_r_, risk cost, which represents the cost of protecting forests against insects, diseases, and wildfire), and the resulting net ecosystem services (NVES) value after accounting for these costs for artificial forests (created during China's national afforestation program) and natural forests in 2014.^a^

				Costs					
		Benefits		*C*_d_	*C*_o_		*C*_r_		
Forest type		VES	timber sales	investments in ecological conservation	land rent	water	management to prevent natural disasters	NVES	Net benefit^b^
Artificial	average (×10^3^ Ɏ ha^−1^·yr^−1^)	46.1	1.4	10.7	0.9	19.9	0.1	14.5	15.9
	total (×10^12^ Ɏ yr^−1^)	3.2	0.1	0.7	0.1	1.4	0.0	1.0	1.1
Natural	average (×10^3^ Ɏ ha^−1^·yr^−1^)	43.2	1.2	7.5	0.7	0.1	0.2	34.8	36.0
	total (×10^12^ Ɏ yr^−1^)	6.4	0.2	1.1	0.1	0.0	0.0	5.1	5.3
Artificial and natural	average (×10^3^ Ɏ ha^−1^·yr^−1^)	44.1	1.3	8.5	0.8	6.4	0.1	28.3	29.5
	total (×10^12^ Ɏ yr^−1^)	9.6	0.3	1.8	0.2	1.4	0.0	6.1	6.4

a The VES was calculated by Niu et al. (2012) and Wang et al. (2017). The timber was considered harvested at an average age of 40 years after afforestation. Timber price was assumed to be an average of Ɏ830 m^−3^ in 2014. Precipitation was based on the average annual value from 1952 to 2014 in China. Potential evapotranspiration was calculated as the average of the estimates from 7 models calibrated for China (Zhang et al. 2016). Investment level represents the average value from 2011 to 2014. In 2014 the yen had a value of ¥6.1428/$US (National Bureau of Statistics 2014).

b The net benefit equals NVES plus the profit from timber sales.

**Figure 1:**
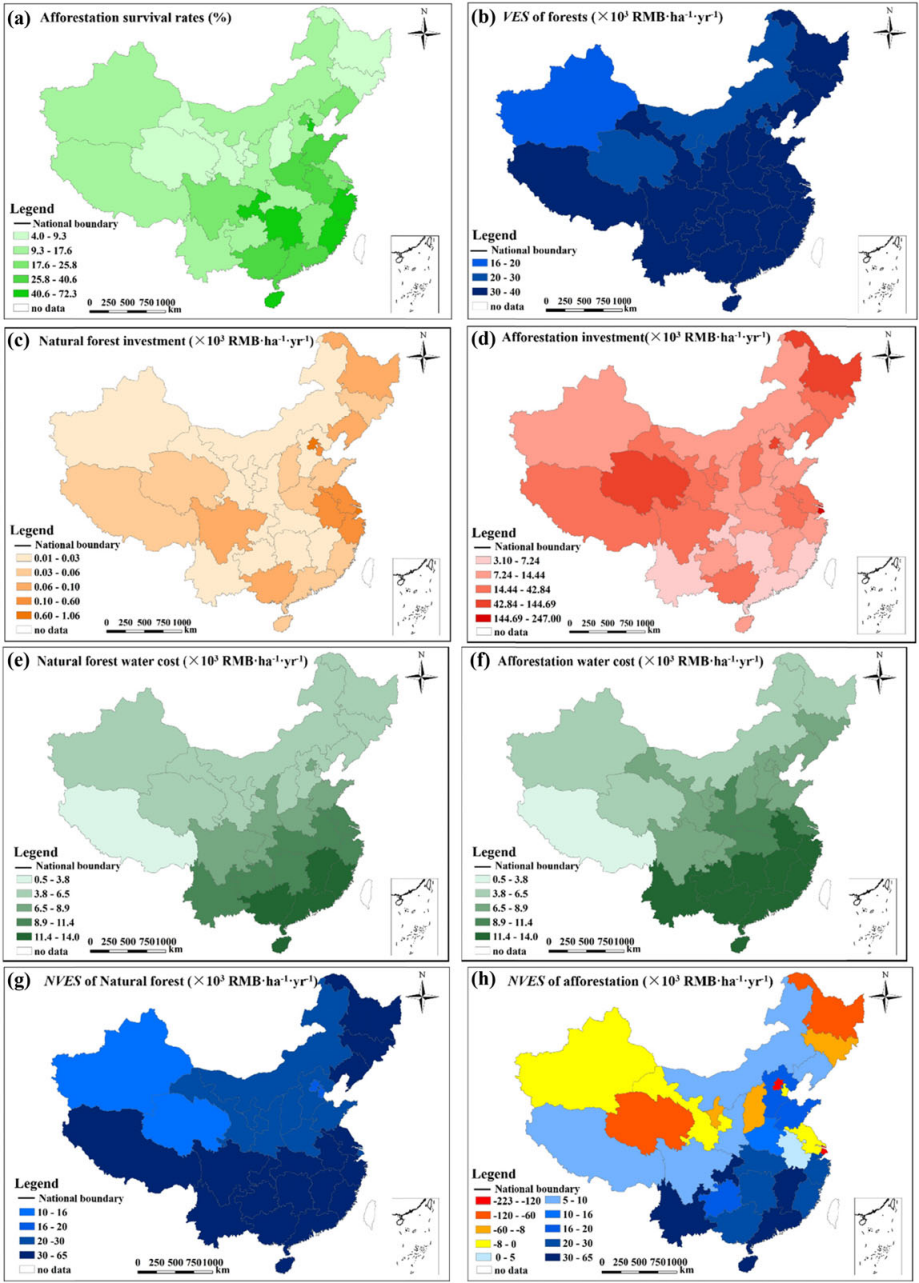
For China's forests established by afforestation (a) survival, (b) value of ecosystem services (VES), (c) investment in natural forest, (d) afforestation investment, (e) natural forest water cost (i.e., the opportunity cost of using water for forests rather than competing uses such as agriculture), (f) afforestation water cost, (g) net value of ecosystem services (NVES) of natural forest, and (h) NVES of afforestation (SFA 1987–2014).

Survival of the artificial forests was greatest in southeastern China, where water is generally abundant and the warm climate favors tree growth (Fig. 1a). The provinces in eastern and southern China had higher VES than those in the other regions (Fig. 1b). In contrast, in the arid northwestern region and the heavily developed Bohai Gulf and Changjiang Estuary regions on the east coast, afforestation produced large negative NVES (Fig. 1h). The NVES for 12 provinces (38.7% of the 31 provinces and provincial‐level regions in China and 40.0% of the total land area) was negative for artificial forest (Fig. 1h).

**Figure 2:**
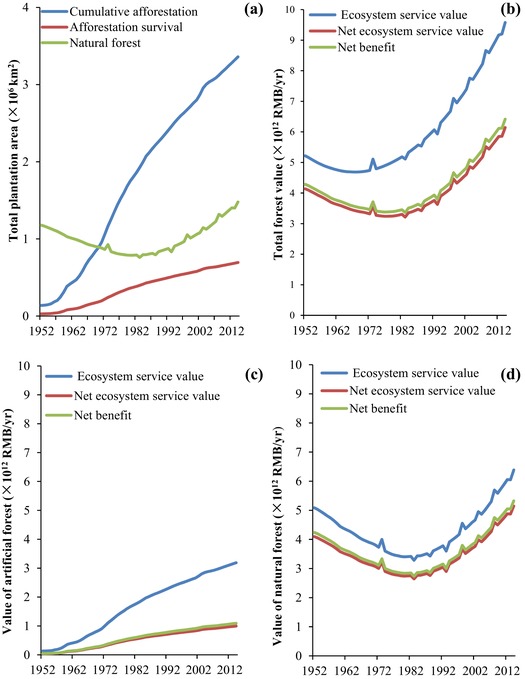
Changes in the forest area and ecosystem service value for natural and artificial forests in China from 1952 to 2014: (a) total forest area and survival of artificial forests, (b‐d) total and net (minus the costs defined in Table 1) forest values, and the net benefit for (b) all forest types combined, (c) artificial forests only, and (d) natural forests only. The net benefit includes the potential value gained from harvesting timber (SFA 1987–2014).

From 1952 to 2014, China's afforestation program affected a total of 3.4 × 10^6^ km^2^, equivalent to 34.8% of the country's land area (Fig. 2a). However, only 837.5 × 10^3^ km^2^ of the artificial forest survived in 2014 (a 20.6% survival rate). The annual NVES of natural forest decreased from Ɏ4.1 × 10^12^ in 1952 to Ɏ2.6 × 10^12^ in 1993 and then increased to Ɏ5.5 × 10^12^ in 2014 (Fig. 2d) as a result of China's natural forest conservation strategy, which was implemented in the late 1980s. However, the annual NVES of artificial forest was Ɏ1.0 × 10^12^ in 2014, which was 68.8% less than the VES value calculated using the traditional approach (Table 1 & Fig. 2c). Adding timber revenues increased the net benefits of afforestation forest and natural forest by 9.6% and 3.5%, respectively, compared with NVES.

## Discussion

Land degradation is a global environmental problem that seriously threatens humans (Sivakumar 2007; D'Odorico et al. 2013). Ecological restoration aims to solve this problem by achieving sustainable socioeconomic development (Tallis et al. 2008). However, ecological restoration efforts still largely rely on a traditional approach that focuses on ecological factors and ignores socioeconomic factors (Meli et al. 2017; Newmark et al. 2017). We found that the net benefits and NVES of creating large‐scale artificial forests through afforestation are considerably smaller than the benefits calculated using the traditional approach (i.e., based solely on VES) after we accounted for the direct, opportunity, and risk costs of this strategy (Table 1). In some cases, the value was even negative (Fig. 1h), which represents a net decrease in benefits. Given the potentially high opportunity costs of any land‐use choice, the difference between benefits and costs is likely to increase when opportunity and other costs are accounted for (Farber et al. 2002). In China, conservation of natural forests seems to be more effective than afforestation; natural forests had a higher NVES.

The traditional way of assessing the VES ignores many costs and therefore does not accurately reflect the real benefits of ecological projects. As a result, it provides inaccurate data to support land‐use planning (Wegner & Pascual 2011). In contrast, NVES‐based analysis improves the accuracy of the analysis because a more complete picture is examined; therefore, it provides better support for land use and ecological restoration planning. It also maximizes the net benefit from such projects by giving managers a chance to correct problems (e.g., negative NVES) before implementing a project (NRC 2005). Although the study of China's forest ecosystems is still in its early stages, our results reveal problems, such as excessive water consumption (i.e., a high water opportunity cost), that require attention. The ability of our method to detect differences among provinces in their VES and NVES clearly demonstrates the importance of basing comparisons of different land conservation and ecological restoration strategies on NVES rather than VES. Therefore, our method can improve planning and policy development because it accounts better for differences in local conditions.

Our results show the importance of basing land‐use policy and assessments of ecosystem services on detailed cost and benefit information, and this will be important anywhere in the world. Land managers must quantify the impacts of changes in land use on the provision of ecosystem services and understand the values and flows of benefits to nearby and distant human populations (Naidoo & Iwamura 2007; Naidoo et al. 2008; Xie et al. 2017). Designing a rational land‐use plan requires that managers account for as many costs as possible to maximize the long‐term net benefits, rather than pursuing a single objective, such as increasing forest cover, that will provide primarily short‐term benefits. The difference in resource endowments among regions leads to different resource prices and different constraints (Crossman et al. 2013). This, in turn, leads to substantial variation in the value of bundles of closely associated ecosystem services provided by any area (Garcia‐Nieto et al. 2013).

Managers must account for the trade‐offs revealed by our results to maximize society's total long‐term benefits, which include the values of goods and ecosystem services, for optimal land use (NRC 2005; Wegner & Pascual 2011; Fu et al. 2013). Because ecological restoration projects are implemented over years or even decades, it is unrealistic to assume that future parameter values will equal the values used at the time of the analysis. It is therefore essential to find ways to forecast the dynamics of benefits and costs when designing ecological restoration plans. To accomplish this, policy makers, economists, and scientists should identify and quantify the key factors that will change over time and then carefully account for the effects of these factors, such as market forces (which determine prices), of regional differences, and of long‐term processes such as climate change in their cost–benefit analysis. This will help them balance the economic and ecological benefits of their plans and correctly grasp the relationship between the short‐term and long‐term benefits.
